# An unusual case of abdominal wall bleeding after renal allograft biopsy

**DOI:** 10.4103/0971-4065.78083

**Published:** 2011

**Authors:** C. G. Koshy, B. R. Chacko, S. Babu, G. Basu, D. Selvaraj, G. T. John

**Affiliations:** Department of Radiology, Christian Medical College, Vellore, India; 1 Department of Nephrology, Christian Medical College, Vellore, India; 2 Department of Vascular Surgery, Christian Medical College, Vellore, India

**Keywords:** Abdominal wall bleeding, angiography, pseudoaneurysm, renal allograft biopsy complication

## Abstract

We report an unusual case of a enlarging anterior abdominal wall hematoma after percutaneous biopsy of a renal allograft. Angiography-directed embolization of the vessels filling the pseudoaneurysm was done and followed up with surgical exploration of the hematoma. In order to avoid this complication, Color Doppler evaluation of the overlying abdominal wall is suggested to look for significant vessels before the biopsy procedure.

## Introduction

Percutaneous renal allograft biopsies are routinely performed. The use of ultrasound guidance and narrow gauge biopsy needles have reduced the chances of significant bleeding. This is a report of an enlarging abdominal wall hematoma that occurred after percutaneous biopsy of a renal allograft. Angiography-directed embolization of the vessels supplying the pseudoaneurysm was done followed by surgical exploration of the hematoma.

## Case Report

A 40-year-old lady who had undergone renal transplantation 4 years ago presented to our hospital with fever, hematuria, nausea, vomiting and diarrhea. She was anemic and thrombocytopenic with graft dysfunction that persisted despite treating a urinary tract infection. The possibilities considered were rejection and hemolytic uremic syndrome.

Percutaneous biopsy of the lower pole of the graft kidney was performed under ultrasound guidance using an 18 G needle and a spring-loaded biopsy device (Monopty, Bard Radiology, Covington, Ga, USA) after normalizing the bleeding parameters.

**Figure 1:**
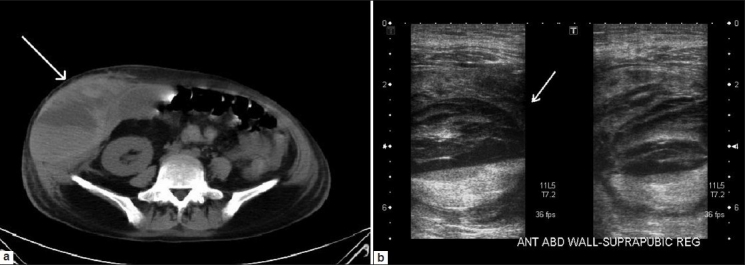
Ultrasound image (a) and computed tomography image (b) showing the hematoma (arrows) within the abdominal wall overlying the graft kidney

A tender swelling developed at the puncture site a few hours after the procedure. Ultrasonography showed a hematoma within the abdominal wall overlying the graft kidney with no perinephric fluid [Figure 1a]. The hematoma increased in size the next day and a non-enhanced CT scan was done [Figure 1b]. The hematocrit further dropped with derangement of the coagulation profile necessitating transfusion with blood and fresh frozen plasma.

**Figure 2:**
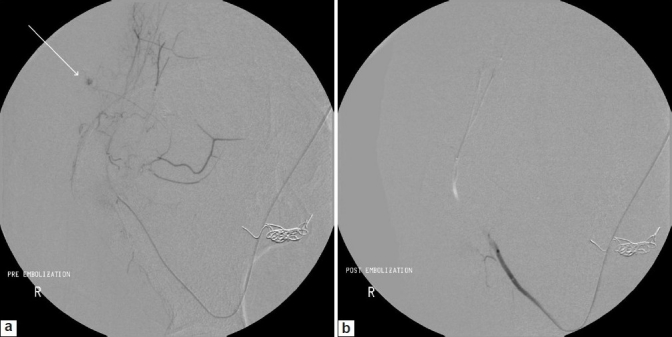
Digital subtraction angiography image showing a pseudoaneurysm (arrow) arising from the ascending branch of the deep circumflex iliac artery. Embolisation coils are seen within the lower pole of the graft kidney. The post-embolisation angiogram (b) showed stasis in the supplying vessel

Digital subtraction angiography showed an arteriovenous fistula in the lower pole of the graft kidney which was embolized with fibered platinum microcoils (Boston Scientific Corp, Watertown, MA, USA) and N-butyl-2-cyanoacrylate (Histacryl glue, B.Braun, Tuttlingen, Germany). Additionally, a pseudoaneurysm arising from the ascending branch of the deep circumflex iliac artery was seen [Figure 2a], which was embolized using gelfoam slurry and polyvinyl alcohol (PVA) particles [Figure 2b]. The inferior epigastric artery could not be demonstrated.

**Figure 3:**
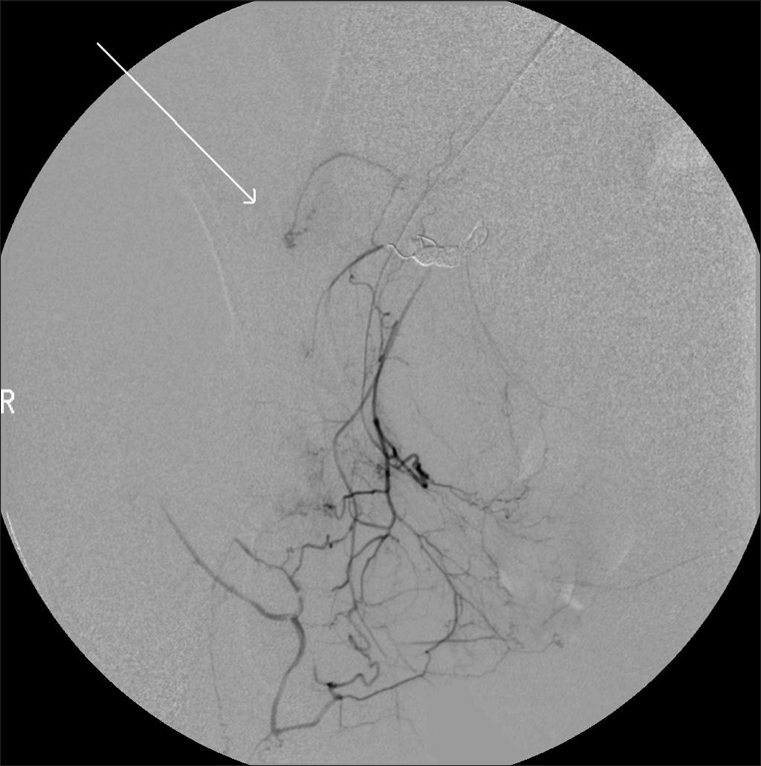
Digital subtraction angiography image of the second session showing the pseudoaneurysm (arrow) being filled by small anastomosing vessels

**Figure 4:**
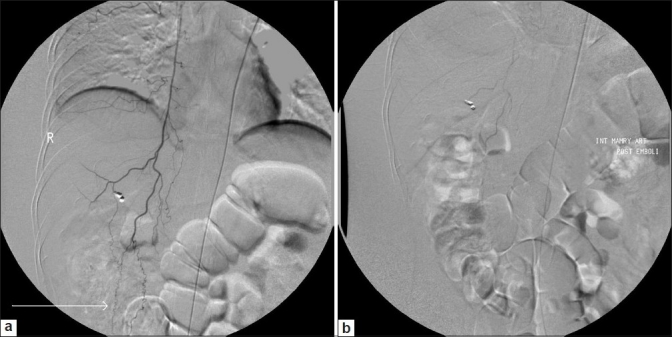
(a) Digital subtraction angiography image showing multiple anastomosing branches (arrow) of the superior epigastric artery anastomosing in the right lower abdomen. Radioopaque cholecystectomy clips are also seen (b) Post embolisation angiogram showing stasis in the superior epigastric artery

Since the hematocrit did not stabilize despite transfusion of blood, platelets and fresh frozen plasma, a repeat angiogram with left common femoral artery approach and selective cannulation of the internal mammary artery was done which revealed persistent opacification of the pseudoaneurysm by collateral vessels in the right iliac fossa [Figure 3]. Using a 4F Glide Headhunter (Cook, Bloomington, USA) catheter and coaxial microcatheter technique, a selective angiogram of the right internal mammary artery was done to demonstrate the superior epigastric artery and its anastomosing vessels branching toward the right lower abdomen [Figure 4a] which were embolized using PVA particles and gelfoam slurry [Figure 4b].

The hematocrit stabilized after this and the hematoma was surgically evacuated due to its large mass effect. A few small bleeding vessels in the wall of the hematoma were cauterized. Repeat CT angiogram was not done in order to avoid contrast-induced nephropathy in an already compromised situation. Clinical improvement was used as a predictor of the resolution of the problem. A follow-up Color Doppler study did not show any vascular lesion in the region of the pseudoaneurysm. The patient was in good health when seen during a follow-up visit 3 months later.

## Discussion

Although the incidence of complications after a percutaneous renal biopsy has decreased with the use of smaller gauge biopsy needles, biopty devices and ultrasound guidance,[1,2] biopsy procedures of renal allografts are known to have a higher incidence of gross hematuria, hematoma formation, hemoperitoneum and arteriovenous fistulae.[3,4] While minor complications generally resolve spontaneously, major complications like acute renal obstruction, arteriovenous fistula, abscess formation and septicemia require further treatment, sometimes even evacuation of the hematoma or nephrectomy.[5]

**Figure 5:**
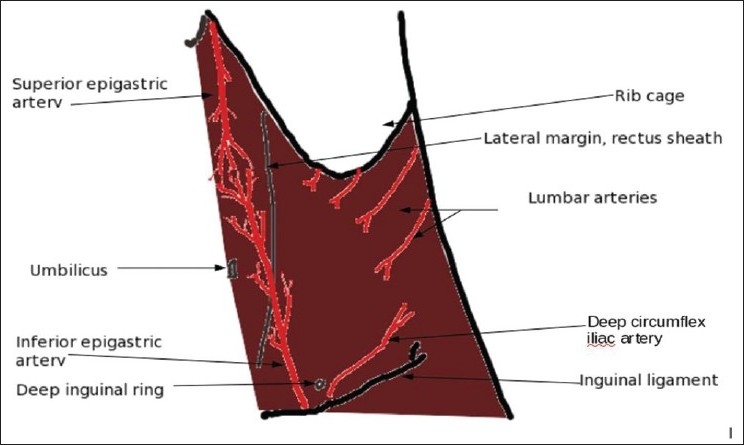
Diagram showing the anastomosing vessels in the anterior abdominal wall

Knowledge of the anterior abdominal wall vasculature and the possible sources of bleeding is useful. The inferior epigastric and the deep circumflex iliac arteries are branches of the external iliac artery, the former being anteromedially oriented and the latter posterolateral. The inferior epigastric artery ascends along the medial margin of the deep inguinal ring, dividing into numerous branches to anastomose with the branches of the superior epigastric artery and the ascending branch of the deep circumflex iliac artery in the anterior abdominal wall[6][Figure 5].

Although the ascending branch of the deep circumflex iliac artery was embolized in the first session, the pseudoaneurysm continued to be filled by collaterals from the superior epigastric artery. Angiography could not demonstrate the inferior epigastric artery probably because of the mass effect of the hematoma on the vessel. In these situations, the treatment options for the pseudoaneurysm include direct compression, direct injection of thrombin, direct or transarterial coil embolization or Histoacryl glue injection. Each option has its advantages and disadvantages and is used depending on the situation, the experience of the operator and the expertise in using them. As this pseudoaneurysm was very small (less than 2-3 mm) and was filling in the delayed phase of the angiography, it was not ideal for embolization with coils. Considering the extra cost involved in using multiple microcatheters for Histoacryl glue injection, we preferred to use PVA and gelfoam particles for embolization. Although resorption of PVA would occur in a few weeks, it would provide occlusion for enough time required for thrombosis of the pseudoaneurysm and the reparative process to occur.[7]

The vessels within the anterior abdominal wall could get injured during invasive procedures and hemostasis is achieved in most cases. There could be a significant bleed from the inferior epigastric artery if there is an underlying coagulopathy and could occur after a biopsy, laparoscopic procedures, paracentesis, surgical trauma or percutaneous drain placement.[8] While a significant bleed from the inferior epigastric artery has been reported after biopsy of an inguinal node[9], it is very rare after a renal allograft biopsy.[10]

In our case, the chronic uremic status and thrombocytopenia could have increased the chances of a significant bleed which might have been avoided if a Color Doppler evaluation of the abdominal wall overlying the graft kidney could have been done before the biopsy. This has been described earlier[11–14] and would help to exclude the presence of significant vessels in the proposed track of the biopsy needle.

## Conclusion

Significant injury to the abdominal wall vessels after a percutaneous renal allograft biopsy is rare and could lead to an enlarging hematoma amidst other complications. The vessels anastomosing in this region are the branches of the inferior and superior epigastric arteries along with the ascending branch of the deep circumflex iliac artery. A simple precaution to prevent a significant bleed would be a Color Doppler evaluation of the abdominal wall overlying the graft kidney, to identify significant vessels in the planned track of the needle.
